# Targeted Gene-Silencing Reveals the Functional Significance of Myocardin Signaling in the Failing Heart

**DOI:** 10.1371/journal.pone.0026392

**Published:** 2011-10-18

**Authors:** Mario Torrado, Raquel Iglesias, Alberto Centeno, Eduardo López, Alexander T. Mikhailov

**Affiliations:** 1 Developmental Biology Group, Institute of Health Sciences, University of La Coruña, La Coruña, Spain; 2 Experimental Surgery Unit, University Hospital Center of La Coruña, La Coruña, Spain; Cardiovascular Research Institute Maastricht, Maastricht University, The Netherlands

## Abstract

**Background:**

Myocardin (MYOCD), a potent transcriptional coactivator of smooth muscle (SM) and cardiac genes, is upregulated in failing myocardium in animal models and human end-stage heart failure (HF). However, the molecular and functional consequences of *myocd* upregulation in HF are still unclear.

**Methodology/Principal Findings:**

The goal of the present study was to investigate if targeted inhibition of upregulated expression of *myocd* could influence failing heart gene expression and function. To this end, we used the doxorubicin (Dox)-induced diastolic HF (DHF) model in neonatal piglets, in which, as we show, not only *myocd* but also *myocd*-dependent SM-marker genes are highly activated in failing left ventricular (LV) myocardium. In this model, intra-myocardial delivery of short-hairpin RNAs, designed to target *myocd* variants expressed in porcine heart, leads on day 2 post-delivery to: (1) a decrease in the activated expression of *myocd* and *myocd*-dependent SM-marker genes in failing myocardium to levels seen in healthy control animals, (2) amelioration of impaired diastolic dysfunction, and (3) higher survival rates of DHF piglets. The posterior restoration of elevated *myocd* expression (on day 7 post-delivery) led to overexpression of *myocd*-dependent SM-marker genes in failing LV-myocardium that was associated with a return to altered diastolic function.

**Conclusions/Significance:**

These data provide the first evidence that a moderate inhibition (*e.g.*, normalization) of the activated MYOCD signaling in the diseased heart may be promising from a therapeutic point of view.

## Introduction

Despite diagnostic and therapeutic advances in clinical cardiology, heart failure (HF), both systolic and diastolic, remains a leading cause of morbidity and mortality in developed countries. The precise stimuli for and mechanisms of ventricular remodeling in acquired HF are not yet clearly delineated. Early studies used a candidate gene approach focused mainly on factors within adrenergic and renin-angiotensin pathways. A recent trend, based on a gene expression topology of the developing and diseased heart, has resulted in the re-interpretation of pathological ventricular remodeling in terms of rearrangement of key gene regulatory networks and downstream signaling pathways that are imbalanced, attenuated, or abnormally activated in failing myocardium [1], [2], [3], [4], [5]. A prominent example of the latter is a serum response factor (SRF)-myocardin (MYOCD) signaling cascade, which is expressed by and modulates gene expression of embryonic, fetal and postnatal cardiomyocytes (reviewed in [6], [7], [8], [9], [10], [11], [12]).

The ability of MYOCD to contribute to heart development and cardiomyocyte differentiation is conserved, although to a different extent, in frogs, chickens, and mammals. Inhibition of endogenous *myocd* expression/function in *Xenopus* [13], [14] and chick [15] embryos is associated with impaired heart development. By contrast, in mouse embryos total knockout [16] or cardio-restricted inactivation [17] of the *myocd* gene does not alter heart development. However, after birth mutant mice with a conditionally inactivated *myocd* gene develop dilated cardiomyopathy accompanied by impaired cardiomyocyte structural organization and severely depressed systolic function. In chimeric *myocd* knockout mice, generated by injection of *myocd* ^−/−^ embryonic stem cells into *myocd* ^+/+^ blastocysts, *myocd* ^−/−^ cells almost completely fail to contribute to formation of ventricular myocardium, although *myocd* ^−/−^-derived myocytes were phenotypically normal [18]. The results suggest that in fetal mouse heart *myocd* is specifically required for functional differentiation of ventricular cardiomyocytes.

Despite its importance in heart development and cardiomyocyte differentiation, *myocd* activation appears to be involved in the adaptive hypertrophic response of the heart during early postnatal development [19] and aging [20]. In fact, *myocd* expression and activity are enhanced in cardiomyocytes upon hypertrophic stimuli [21], [22], [23]. In addition, *in vitro* forced expression of *myocd* in mouse neonatal cardiomyocytes increases cell size and activates transcription of established *myocd* targets, such as smooth muscle (SM) α-actin (ACTA2) and SM myosin heavy chain (MYH11), along with a set of cardiac hypertrophy-associated genes [21]. *Myocd* inactivation, by dominant-negative mutant [21] or interfering RNA [22], inhibits agonist-induced hypertrophy in cardiomyocytes, but does not provoke either disruption of sarcomeric structure or cardiomyocyte atrophy. In neonatal piglet heart, *in vivo* forced *myocd* expression upregulates genes for SM22α/transgelin (TAGLN) and fetal muscle light chain 3f myosin associated with transiently impaired systolic function [24]. In postnatal mouse heart, conditional knockdown of *myocd* leads to the rapid-onset of HF due to dilated cardiomyopathy which is associated with attenuated expression of SRF/MYOCD-regulated cardiac genes and activation of pro-apoptotic factors in failing myocardium [17]. Taken together, these observations suggested that both MYOCD redundancy and deficiency in postnatal cardiomyocytes affect cardiac performance.

In addition to its role in adaptive gene expression and maintenance of cardiac function, *myocd* has also been implicated in the response of the postnatal/adult heart to pathological stresses during hypertrophic remodeling [21], [25], cardiomyopathic progression [26] and at end-stage HF [19], [21]. All of these conditions are characterized by upregulation of *myocd* expression in failing left ventricular (LV) myocardium. In addition, *myocd* targets, such as ACTA2 [27], [28], SM-calponin (CNN1), and MYH11 [29], are also upregulated in failing myocardium in both animal models and patients. Although this correlative evidence links MYOCD signaling to acquired pathological conditions, the role that *myocd* gene activation plays in HF conditions *in vivo* has yet to be determined.

A provocative hypothesis was that *myocd* overexpression might represent a maladaptive response of ventricular myocardium to pathological remodeling [8], [24]. The goal of the present study was, therefore, to investigate if targeted inhibition of upregulated expression of *myocd* in failing ventricular myocardium could normalize dysregulated MYOCD signaling pathways and restore, at least in part, impaired cardiac function. To this end, we used the doxorubicin (Dox)-induced diastolic HF (DHF) model in neonatal piglets, in which upregulation of *myocd* was established as a HF-related feature [19]. In the present work, we extend these results demonstrating that not only *myocd* but also *myocd*-dependent SM-marker genes are highly activated in failing LV-myocardium of Dox-injected piglets.

*In vivo* silencing of endogenous *myocd* expression by short-hairpin (sh) interfering RNAs at advanced stages of DHF in piglets resulted in downregulation of *myocd*-dependent SM-gene expression in failing myocardium. Such adjusting of MYOCD and SM-target levels to the scores measured in non-failing controls resulted in restoring diastolic function and extending the survival of failing animals. These data provide the first evidence that a moderate inhibition (*e.g.*, normalization) of activated MYOCD signaling in the diseased heart may be promising from a therapeutic point of view.

## Materials and Methods

### Animals and experimental design

**Figure 1:**
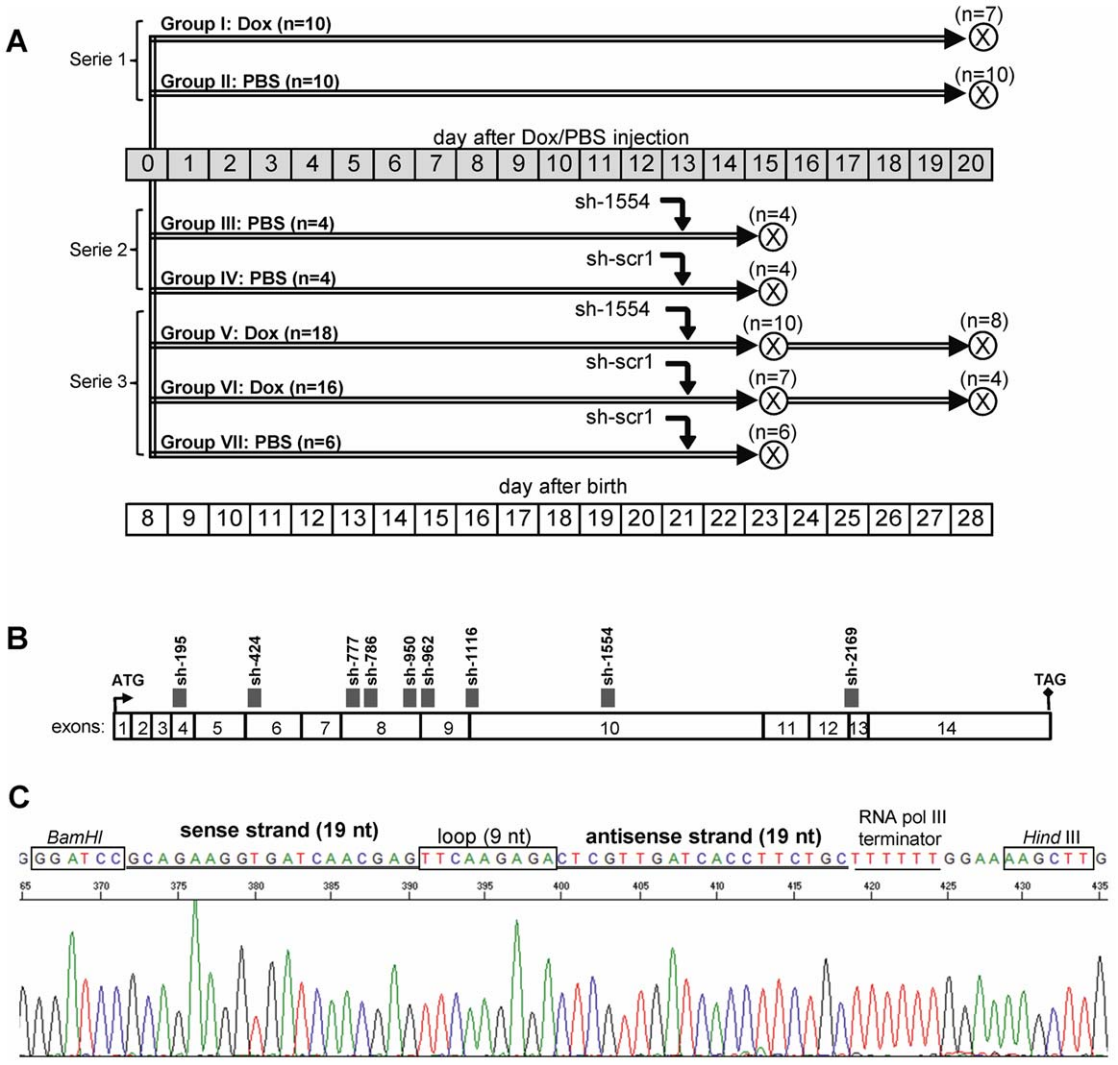
Experimental designs. (A) Eight-day-old piglets were randomized in seven groups, assigned to receive doxorubicin (Dox) or isotonic saline vehicle (PBS) and used in the following experimental series: (1) DHF model (Group I and II), (2) *myocd* silencing in non-failing piglets (Group III and IV), and (3) *myocd* silencing in failing DHF piglets (Group V, VI, VII). Black arrows – intramyocardial delivery of *myocd*-silencing (sh-1554) or scrambled (sh-scr1) vectors. X-crossed circle – day of sacrifice. (B) Schematic localization of short hairpin (sh-195–sh-2311) target sites (grey) within the coding region of pig *myocd* (GenBank accession number NM_213745). Translation initiation (ATG) and termination (TAG) codons are shown. (C) Short hairpin template design. The 5′ ends of the two strands (sense-antisense) are non-complementary and form the *Bam*H I and *Hind* III restriction site overhangs. The short hairpin inserts and their orientation were confirmed by vector sequencing (as exampled here with the sh1554 vector).

Early neonatal “Large White” piglets were obtained from a local commercial breeder (La Coruña, Spain), maintained in a conventional Nürtinger nursery system for days 8 after birth, and randomized in seven groups according to experimental design (Fig. 1A). A diastolic heart failure (DHF) model was established in 8-day-old neonatal piglets by an i.v. injection of cardio-toxic agent, Doxorubicin (Dox; Sigma, Madrid, Spain) of a single dose of 2.0 mg/kg as previously described [30]. Animals injected with normal isotonic saline (PBS) were used as controls. Some Dox-injected (Group I) and PBS-injected (Group II) animals were not additionally manipulated, but others were subjected to intramyocardial delivery of plasmids expressing either short hairpin (sh) *myocd*-silencing (Group III and V) or sh-scrambled (Group IV, VI, and VII) vectors before the planned end of the study. At the end of each experimental run (see Fig. 1A), ECGs and cardiac output (PiCCO device, Pulsion AG, Germany) were monitored in closed-chest piglets, while measurements of LV end-systolic (ESP) and end-diastolic pressure (EDP) were performed in open-chest piglets using a Dräger UM3.1 pressure transducer and a recording device (Drägerwerk AG, Germany) as described in [30]. Then piglets were euthanized to harvest cardiac tissues for RNA and protein isolation. Piglets were used in accordance with the European Commission Directive 86/609/EEC and all protocols were approved by the Institutional Animal Care and Use Ethics Committee (permit number: PI 1–2008; University of La Coruña, La Coruña, Spain).

### RNA isolation

For total RNA isolation, deep-frozen tissue/cell samples were directly disrupted in RLT buffer (Qiagen, Madrid, Spain) using a high-speed rotor-stator homogenizer (Ultra-Turrax T8, Germany), digested with Proteinase K (Qiagen), loaded onto RNeasy Mini/Midi columns (Qiagen), subjected to on-column digestion of DNA with RNase-free DNase (Qiagen), and processed in accordance with the manufacturer's recommendations. Resulting RNA preparations were ethanol-precipitated, resolved in RNase-free H_2_O, and kept at −80°C. RNA yield and purity was determined spectrophotometrically at 260–280 nm and RNA integrity was verified by running samples on 1.2% agarose gels and staining with ethidium bromide.

### Microarray

Total RNAs isolated from LV biopsies of three failing (i.e., Dox-injected) and three non-failing (i.e., PBS-injected) piglets were independently hybridized on the Affymetrix GeneChip® Porcine Genome Array (Affymetrix, Santa Clara, US), which contains 23,937 probe sets interrogating 23,256 transcripts and representing 20,201 genes of *S. scrofa*. Sample processing, array hybridization, scanning, and quantification were performed at the Affymetrix Service Provider and Core Facility, “KFB - Center of Excellence for Fluorescent Bioanalytics” (University of Regensburg, Regensburg, Germany; www.kfb-regensburg.de). Genes were considered to be differentially expressed if a change of at least 2-fold was observed for evaluated (failing *versus* non-failing) comparisons at a p-value cutoff of 0.05. The microarray data have been deposited in the NCBI's Gene Expression Omnibus (GEO; [31]) and are accessible through GEO Series accession number GSE30110 (http://www.ncbi.nlm.nih.gov/geo).

### Design and generation of short-hairpin RNA plasmids

Nine short-hairpin (sh) 19-mer sequences located throughout the full-length of the porcine *myocd-B* mRNA (Fig. 1B) were designed using the basic tools [32], [33]. The sh-RNAs were designed to target sites devoid of single nucleotide polymorphisms, and correspond to splice *myocd-A/B* variants identified in pig myocardium [19], [24]. Each oligonucleotide pair included (Fig. 1C): a *Bam*HI overhang on the 5′ end of the duplex, the 19 nucleotides of the sh-RNA sense strand, a loop sequence, the 19 nucleotides of the RNA antisense strand, a Pol III termination site of 6 consecutive thymidine residues, and a *Hind* III overhang on the 3′ end of the duplex. The scrambled sh-RNA sequence 1 (sh-scr1, see Table 1) was constructed in an identical manner; the sh-scr2 was from Ambion. Annealed oligonucleotides encoding sh-RNAs were cloned in a p*Silencer* 2.1-U6 puro vector in accordance with the manufacturer's protocol (Ambion, Cambridgeshire, UK). Resulting plasmids were verified by sequencing as a service provided by Secugen (Madrid, Spain). For transfections, plasmids were purified using a PureLink HiPure plasmid filter purification kit (Invitrogen, Barcelona, Spain) according to the manufacturer's protocol.

**Table 1:** Oligonucleotides used for constructing short-hairpin RNAs.

Target region	Nucleotide sequence
195-S	GATCCGCACAAGGTCAGAAACAGGTTCAAGAGACCTGTTTCTGACCTTGTGCTTTTTTGGAAA
195-AS	AGCTTTTCCAAAAAAGCACAAGGTCAGAAACAGGTCTCTTGAACCTGTTTCTGACCTTGTGCG
424-S	GATCCGTGAGTCTCTCCAAATCAGTTCAAGAGACTGATTTGGAGAGACTCACTTTTTTGGAAA
424-AS	AGCTTTTCCAAAAAAGTGAGTCTCTCCAAATCAGTCTCTTGAACTGATTTGGAGAGACTCACG
777-S	GATCCGGTGAAGAAGCTCAAGTACTTCAAGAGAGTACTTGAGCTTCTTCACCTTTTTTGGAAA
777-AS	AGCTTTTCCAAAAAAGGTGAAGAAGCTCAAGTACTCTCTTGAAGTACTTGAGCTTCTTCACCG
786-S	GATCCGCTCAAGTACCACCAGTATTTCAAGAGAATACTGGTGGTACTTGAGCTTTTTTGGAAA
786-AS	AGCTTTTCCAAAAAAGCTCAAGTACCACCAGTATTCTCTTGAAATACTGGTGGTACTTGAGCG
950-S	GATCCGCTCAGCTCAAGGAACCAATTCAAGAGATTGGTTCCTTGAGCTGAGCTTTTTTGGAAA
950-AS	AGCTTTTCCAAAAAAGCTCAGCTCAAGGAACCAATCTCTTGAATTGGTTCCTTGAGCTGAGCG
962-S	GATCCGGAACCAAATGAACAGATGTTCAAGAGACATCTGTTCATTTGGTTCCTTTTTTGGAAA
962-AS	AGCTTTTCCAAAAAAGGAACCAAATGAACAGATGTCTCTTGAACATCTGTTCATTTGGTTCCG
1116-S	GATCCGGTCTCCGAGTTAAGACAATTCAAGAGATTGTCTTAACTCGGAGACCTTTTTTGGAAA
1116-AS	AGCTTTTCCAAAAAAGGTCTCCGAGTTAAGACAATCTCTTGAATTGTCTTAACTCGGAGACCG
1554-S	GATCCGCAGAAGGTGATCAACGAGTTCAAGAGACTCGTTGATCACCTTCTGCTTTTTTGGAAA
1554-AS	AGCTTTTCCAAAAAAGCAGAAGGTGATCAACGAGTCTCTTGAACTCGTTGATCACCTTCTGCG
2169-S	GATCCGCAGCAAATGACTCGGAGTTTCAAGAGAACTCCGAGTCATTTGCTGCTTTTTTGGAAA
2169-AS	AGCTTTTCCAAAAAAGCAGCAAATGACTCGGAGTTCTCTTGAAACTCCGAGTCATTTGCTGCG
SCR1-S	GATCCGCCCGCAAATCGTCTAATCTTCAAGAGAGATTAGACGATTTGCGGGCTTTTTTGGAAA
SCR1-AS	AGCTTTTCCAAAAAAGCCCGCAAATCGTCTAATCTCTCTTGAAGATTAGACGATTTGCGGGCG

S - sense, AS – antisense.

### Cell culture and transfection *in vitro*

COS-7 and porcine aortic smooth muscle cells (PAOSMCs) were purchased from the European Collection of Cell Cultures (ECACC; Salisbury, UK). COS-7 cells were cultured in Dulbecco's modified Eagle's Medium (Gibco, Barcelona, Spain) supplemented with 10% fetal bovine serum (FBS) and penicillin–streptomycin–glutamine (Gibco) under standard tissue culture conditions at 37°C. Cells were trypsinized at 70–80% confluence, cell numbers were determined using an automated cell counter (Countess, Invitrogen, Barcelona, Spain) and 80,000 cells were plated in each of the 12-well plates and allowed to attach overnight. Porcine *myocd* and *ankrd1* (ankyrin repeat domain 1 protein) were amplified from piglet LV oligo-dT-primed cDNA, cloned into p3XFLAG-CMV-14 (Sigma, Madrid, Spain) vectors, verified by sequencing, and purified as previously described [24], [34]. Each batch of COS-7 cells was co-transfected with plasmids expressing: (1) either p*Silencer* 2.1-U6 puro *myocd* sh-RNA or p*Silencer* 2.1-U6 puro scramble sh-RNA vectors, (2) FLAG-tagged pig *myocd-B*, and (3) FLAG-tagged pig *ankrd1* constructions. All transfections were carried out with Lipofectamine LTX and PLUS Reagents (Invitrogen, Barcelona, Spain) following the manufacturer's instructions. For each combination of plasmids, two separate transfection assays were employed, and in each assay, transfections were performed in duplicate. Transfected cells were cultured for 9 hours, then culture media were replaced with fresh media supplemented with 10% FBS. The cells were harvested at 24–48 hours after transfection and processed for RNA and protein extraction [34], [35]. PAOSMCs were cultivated in accordance with the ECACC recommendations (Cell Applications Inc., San Diego, US) in P311–500 growth medium (Sigma, Madrid, Spain), until 80% confluency was reached, and then transfected with *myocd*-silencing or scramble sh-vectors essentially as described above for COS-7 cells. The sh-RNA-expressing populations were selected with puromycin (Invitrogen, Barcelona, Spain) at the concentration of 1.0 µg/ml. Stable transfectants were grown in the presence of 1.0 µg/ml of puromycin for 6 days and then processed for RNA and protein extraction.

### Intramyocardial transfection *in vivo*

In accordance with experimental design (see Fig. 1), naked plasmids expressing sh-*myocd*-1554 or sh-scr1 constructions were delivered into the LV free wall (LVFW) of Dox- and PBS-injected piglets. Transcatheter, intramyocardial delivery was performed using a protocol developed and validated in our laboratory [24]. Briefly, under anesthesia and automatic ventilatory support, a fiber-optic catheter (Cardio-Optics Inc., Boulder, US) and endoscopic cannula were introduced into the left chest cavity. Then, the endoscopic needle was introduced into the cannula, and 3–4 intramyocardial injections were performed in the ventro-lateral area of the LVFW under video-assisted real-time visualization. On the 2nd and 7th day post-delivery, ECG, LV-ESP and LV-EDP parameters were measured in piglets as described above. All these procedures were conducted by personnel blinded to the experimental design. Then animals were euthanized and the hearts were rapidly excised, weighed, and photographed. The ventral LVFW of each heart was sectioned into 3–4 regions (corresponding to the injection sites) which were then assayed individually for DNA, RNA and protein isolation.

### Semiquantitative PCR

To study the level and persistence of plasmid DNA in transfected piglet myocardium, total DNAs were isolated from LV samples of Dox-injected piglets on day 2 and 7 post-delivery using the Qiagen DNeasy Kit (Qiagen, Madrid, Spain). The level of plasmid DNA in extracted total DNAs was quantified using semiquantitative PCR with primers surrounding the sh-insert in the p*Silencer* 2.1-U6 puro vector that amplify a fragment of predicted size (532 bp; see Table 2). PCR was performed in a Biometra II system (Gottingen, Germany) using the U2 gene as an internal standard. The amount of DNA and the number of cycles were varied for each primer pair to ensure amplification within the linear phase. PCR products were visualized on 2% agarose gels by ethidium bromide staining and relative band intensity was estimated by densitometry (VersaDoc 1000) and Quantity One software (Bio-Rad, Hercules, US).

**Table 2:** Primers used in this study.

Primer	Target	Sequence (5′-3′)	PCR product	Application
330	*U2*	TCGCTTCTCGGCCTTTTGGCT		
331	*U2*	GTACTGCAATACCAGGTCGATG	330-331: 168 bp	PCR
356	*pSilencer*	AGGCGATTAAGTTGGGTAACGC		
357	*pSilencer*	GGAATTAATACGACTCACTATAGGGAGA	356-357: 532 bp	PCR
271	*myocd-B*	CAGGTGTGCAGCAAAAGATGGT		
270	*myocd*	CCGAAACTGCTGAGGCTGACT	271-270:105 bp	qPCR
272	*myocd-A*	GGTGTGCACTGCACAGATGGT		
270	*myocd*	CCGAAACTGCTGAGGCTGACT	272-270: 104 bp	qPCR
64	*rpl19*	CTGCTCAGAAGATACCGTGAAT		
206	*rpl19*	GCTTGTGGATGTGCTCCATGA	64-206:121 bp	qPCR
341	*srf*	AGCCTCATGCAGCTGCCTACTA		
340	*srf*	GTGCACCTGTATGGCCTGTAC	341-340: 87 bp	qPCR
349	*myh11*	AAACTGCAGGCTCAGATGAAGGA		
348	*myh11*	TGGCTTTCTTCTCATTCTCTTTGG	349-348:103 bp	qPCR
82	*nppb*	GCTCCTGCTCCTGTTCTTGCA		
251	*nppb*	GGTCCAGCAGCTCCTGTATC	82-251:107 bp	qPCR

*pSilencer* - pSilencer 2.1-U6 puro vector; *nppb* – gene coding for natriuretic peptide B.

### Quantitative real-time RT-PCR

Four µg of RNA were reverse transcribed using SuperScript III reverse transcriptase (Invitrogen, Barcelona, Spain) and oligo-dT primer according to the manufacturer's instructions. Two-step quantitative real-time RT-PCR (qPCR) was performed on a Bio-Rad IQ5 detection system (Bio-Rad, Hercules, US) with SYBR Green I mix following conditions previously described [24], [34]. The reference *rpl19* (ribosomal protein L19) gene was amplified to normalize expression. For each RNA sample, genomic DNA contamination was determined by PCR on a no-RT control for the *rpl19* gene. Within each experiment, PCR reactions were done in duplicate. Each PCR reaction was evaluated using melting curve analysis and checking the PCR products on 8% SYBR Green-stained polyacrylamide gels. Fold changes were calculated using the C_T_ method. Data were analyzed using IQ5 optical system software 2.0 and C_T_ comparative analysis. For primer sequences used in qPCR analysis, please see Table 2.

### Antibodies

Rabbit polyclonal antibodies to transgelin (TAGLN; ab14106), phospholamban (PLN; ab15000), SM-α-actin (ACTA2; ab5694), SM-myosin heavy chain 2 (MYH11; ab53219), SM-calponin (CNN1; ab46794), cardiac troponin I (TNNI; ab47003), cardiac calsequestrin-2 (CASQ2; ab3516), and glyceraldehyde 3 phosphate dehydrogenase (GAPDH; ab9485) were purchased from Abcam (Cambridge, UK). Mouse polyclonal antibodies to SM-γ-actin 2 (ACTG2; abA01) were purchased from Novus Biologicals (Litteton, CO, US). Rabbit polyclonal antibodies to MYOCD (marked by us as AB531; [24]) were generated by Davids Biotechnologie (Regenburg, Germany) using the recombinant TAD-containing fragment of porcine MYOCD as immunogen. The AB531 antibodies were shown to be specific for both MYOCD-A (minor) and MYOCD-B (major) variants identified to date in pig cardiac and SM-containing tissues [24]. Mouse monoclonal anti-FLAG antibodies and secondary peroxidase-conjugated anti-rabbit and anti-mouse IgG (Fab-specific) antibodies were purchased from Sigma (Madrid, Spain).

### SDS-PAGE and Western blotting

Tissue/cell samples were homogenized and solubilized in standard 2× Laemmli buffer (Invitrogen, Barcelona, Spain) supplemented with complete protease inhibitor cocktail (Roche, Madrid, Spain) as previously described [30], [35]. Following centrifugation at 20,000 g for 30 minutes, the concentration of supernatant proteins was analyzed using the Bio-Rad *DC* Protein Assay Kit (Bio-Rad, Hercules, US) according to the manufacturer's protocol. All samples were stored at −80°C before electrophoresis. Protein supernatants (5–10 µg/run or 25–30 µg/run for MYOCD detection) were subjected to SDS-PAGE (Mini-Protean-III, Bio-Rad, Hercules, US), stained with Coomassie R-250 (Merck, Barcelona, Spain) or blotted onto PVDF-membranes (Hybond-P, Amersham Biosciences, Barcelona, Spain). Molecular weight (MW) standards (Precision Plus Protein WesternC Standards from Bio-Rad or SeeBlue Plus2 Pre-Stained Standard from Invitrogen) were included on each gel. Blots were probed with the antibodies indicated above and visualized by the Super-Signal West Pico chemiluminescent substrate (Pierce Biotechnology, Madrid, Spain) as described [30]. Equivalence of protein loading was confirmed by Amido-Black (Merck, Barcelona, Spain) staining of blots after immunodetection. Quantification of Western blot signals was obtained by using a Bio-Rad GS800 calibrated densitometer with Quantity One software.

### Statistics

Results are expressed as mean±SEM. Statistical significance was evaluated by Student's *t*-test. Statistical analyses were performed with SPSS 13 software. A value of p≤0.05 was considered statistically significant.

## Results

### The myocardin-dependent smooth-muscle gene program is activated in diastolic failing heart

Neonatal piglets injected with Dox (see Fig. 1; Group I) develop a cardiotoxic cardiomyopathy that rapidly progresses to diastolic dysfunction with elevated filling pressures (measured as LV-EDP) and ECG abnormalities such as ST/T wave depression and low-voltage QRS complexes. LV-ESP was slightly lower (with preserved cardiac output index) in Dox-injected compared to PBS-injected piglets (data not shown). In addition, experimental piglets showed a substantial decrease in survival between 12 and 20 days after Dox-injection ([30] and this work, see Fig. 1A, Series 1). In the LV myocardium 20 days after Dox administration, DNA microarray analysis revealed a high upregulation of established HF markers/risk factors, with a significant fold change ranging from 4.06 (alpha-2-macroglobulin) to 27.81 (resistin) in Dox-injected as compared to PBS-injected piglets (Table 3). Detailed results of microarray analysis will be reported elsewhere.

**Table 3:** Upregulation of HF markers/risk factors in the piglet DHF model.

Gene coding for	Gene symbol	Fold change	p-value	Cardiac overexpression	Reference
Resistin	RETN	27.81	0.01	maladaptive	[59], [60]
Brain-derived neutrophic factor	BDNF	18.26	0.04	adaptive	[61]
S100 calcium binding protein A1	S100A1	15.40	0.02	adaptive	[62]
Brain natriuretic peptide	BNP	12.77	0.05	adaptive	[63], [64]
Matrix metallopeptidase 9	MMP9	7.63	0.01	maladaptive	[65]
Tumor necrosis factor-alpha	TNFα	6.30	0.01	risk factor of HF	[66]
Alpha-2-macroglobulin	α2M	4.06	0.01	marker of HF	[67]

**Figure 2:**
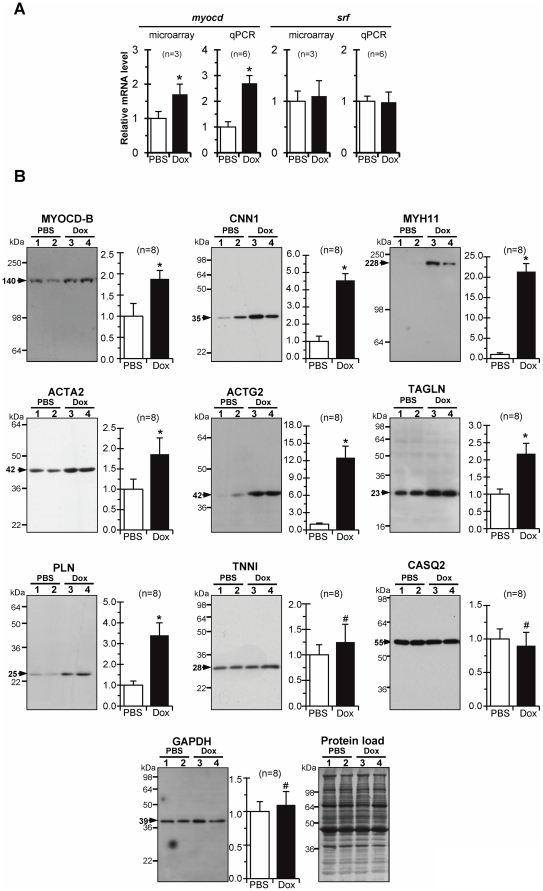
Expression of myocardin and SM-marker genes is activated in diastolic failing heart. (A) Average relative *myocd* and *srf* mRNA levels in failing (Dox) *versus* non-failing (PBS) LV-myocardium samples, as revealed by microarray and qPCR analyses. (B) Representative Western blots and an average fold increase in MYOCD and SM-marker protein levels in failing (Dox) over non-failing (PBS) LV-myocardium samples. Black arrows - MW values (kDa) of the bands detected. Protein load (Amido-Black stained blot after immunodetection). *p≤0.05.

*Myocd* mRNA levels, measured by microarray followed by qPCR analyses, were 2-3-fold higher in failing *versus* non-failing LV myocardium, whereas *srf* expression levels remained unchanged (Fig. 2A). At the protein level (Fig. 2B), a more than twofold increase in MYOCD was observed in Dox-injected animals compared to controls. As expected [24], AB531 anti-MYOCD antibodies revealed a large MYOCD-B variant in LV-myocardium samples (with the MYOCD-A level being generally under Western blot sensitivity limits).

This upregulation of *myocd* expression, at both the mRNA and protein levels, was associated with increased protein expression of MYOCD/SRF downstream SM-targets (CNN1, MYH11, ACTA2, ACTG2, and TAGLN) in failing *versus* non-failing myocardium. In addition, the relative level of phospholamban (PLN - a putative *myocd* target; see [36], [37]) also increased in failing myocardium. The amounts of CASQ2 and TNNI (key regulatory proteins of cardiac muscle contraction and relaxation) were not significantly changed in the LV-myocardium of Dox-injected compared to PBS-injected animals. In addition, as revealed by microarray analysis, the expression of genes encoding cardiac muscle alpha actin (ACTC1), beta-myosin heavy chain (MYH7), and myosin light chain 2 ventricular (MLC2v) was not affected in Dox-injected animals.

These results indicated that the activation of *myocd* and SM-marker genes is among the prominent features of failing myocardium and, therefore, the reduction of *myocd* expression could be an approach that would allow us to specifically target the expression of SM-marker genes in our model of DHF-like syndrome.

### Plasmid-based myocardin-specific short-hairpins effectively inhibit myocardin gene expression in cell-based and *in vivo* assays

**Figure 3:**
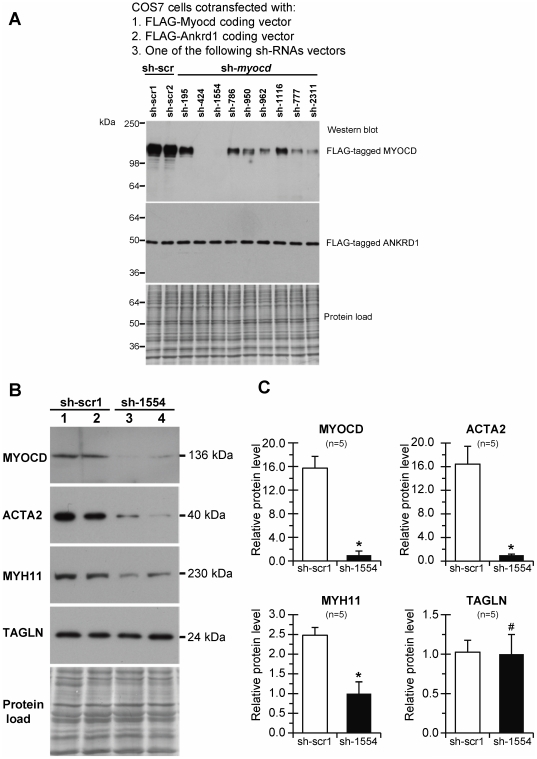
Selection of effective short hairpin constructs against pig myocardin in cell-based assays. (A) Total protein lysates from COS-7 cells co-transfected with FLAG-tagged MYOCD, FLAG-tagged ANKRD1 and indicated sh-*myocd* (sh-195–sh-2311) or scrambled (sh-scr1, sh-scr2) vectors were electrophoresed and immunoblotted with anti-FLAG antibodies. Protein load – Amido-Black stained blot after immunodetection. 36–250 – MW values, kDa. (B) Total protein lysates from PAOSMCs permanently transfected with sh-scr1 (lane 1, 2) or sh-1554 (lane 3, 4) vectors were screened for MYOCD and SM-marker proteins by immunoblotting. MW values (kDa) of the bands detected are shown. Protein load – Amido-Black stained blot after immunodetection. (C) Average fold decrease in levels of MYOCD and SM-marker (ACTA2, MYH11, and TAGLN) proteins in sh-1554-transfected *versus* sh-scr1-transfected PAOSMCs. *p≤0.05.

Nine sh-RNAs were designed against the porcine *myocd-B* RNA. Effects of plasmid-based sh-*myocd* vectors on *myocd* expression were first tested in COS7 cells which did not express the endogenous *myocd* gene. Each batch of COS7 cells was co-transfected with the three vectors indicated (Fig. 3A) and the level of FLAG-tagged MYOCD protein was determined in total cell lysates by Western blot with monoclonal anti-FLAG antibodies. Although to varying degrees, all sh-*myocd* vectors were able to reduce MYOCD protein levels as compared to sh-scrambled (sh-scr) controls. FLAG-tagged ANKRD1 levels were uniform in all samples, indicating efficient transfection and the absence of nonspecific inhibitory or toxic effects on overall transgene expression. Two sh-*myocd* vectors (sh-424 and sh-1554) provoked a nearly complete inhibition of MYOCD protein expression in co-transfected COS7 cells.

The inhibitory efficiency of these sh-*myocd* vectors was then evaluated by using a primary PAOSMC line, because we found that these proliferating cells exhibit a proper expression of *myocd* mRNA and protein at levels sufficient for analysis. Results of transient transfection of PAOSMCs revealed that sh-1554 exhibits more inhibitory activity on *myocd* expression than sh-424 (data not shown). The sh-1554 vector was therefore used for permanent transfection of PAOSMCs. Stable sh-1554 and sh-scr1 transfectants were screened for MYOCD and its SM-target proteins by immunoblotting. As shown in Fig. 3B (lane 3), one of the stable *myocd* sh-1554 transfectants displayed a nearly complete knockdown of MYOCD protein expression. Although none of the sh-1554 transfectants showed complete MYOCD depletion, the average inhibition of MYOCD protein expression was more than 80% from the sh-scr1 transfectant level. This MYOCD inhibition was associated with decreased levels of ACTA2 (the average inhibition – 88%) and MYH11 (the average inhibition – 60%) proteins in sh-1554 *versus* sh-scr1 transfectants, being expression of TAGLN unaffected (Fig. 3B, C).

**Figure 4:**
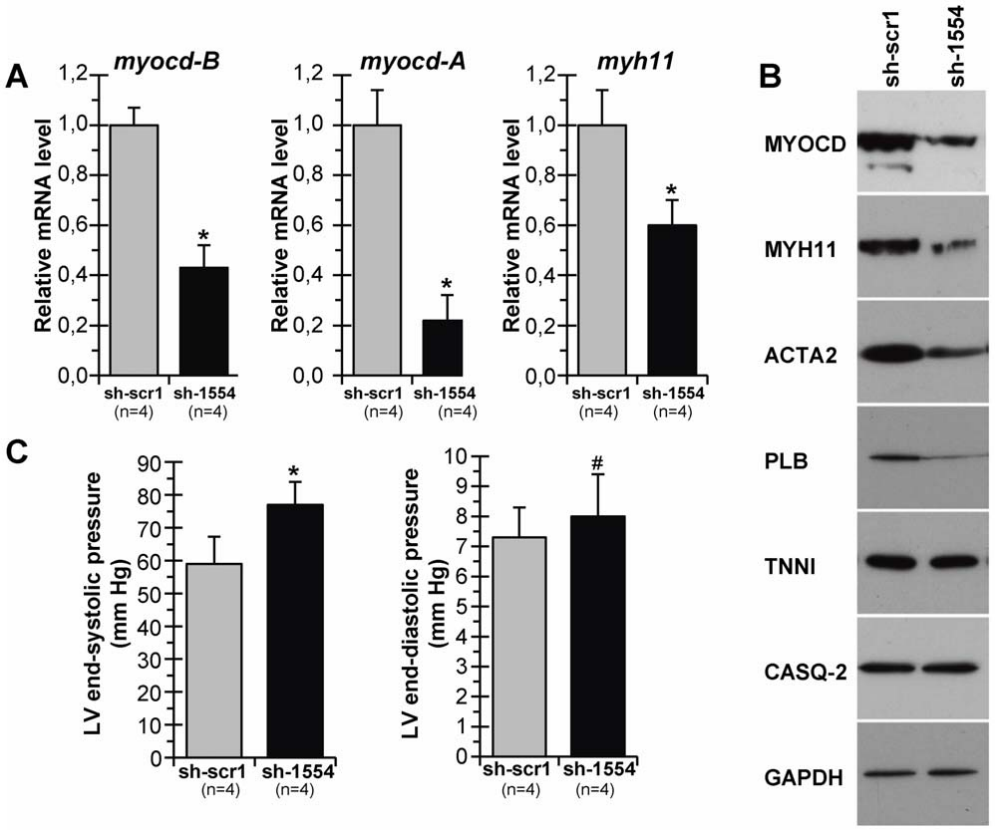
Silencing of myocardin expression in non-failing piglet myocardium *in vivo*. Healthy PBS-injected piglets (see Fig. 1A) were intramyocardially transfected with *myocd*-silencing (sh-1554) or control scrambled (sh-scr1) vectors. (A) Levels of *myocd-A*, *myocd-B* and *myh11* mRNAs detected by qPCR. (B) Representative Western blots of MYOCD, MYH11, ACTA2, PLN, TNNI3, CASQ2, and GAPDH. (C) LV-ESP and LV-EDP values in sh-scr1- and sh-1554-transfected animals. *p≤0.05.

The sh-1554 vector was used in downstream experiments on *myocd* silencing in non-failing piglet heart *in vivo*. To this end, PBS-injected piglets (see Fig. 1A) were intramyocardially transfected with sh-1554 (Group III) or sh-scr1 (Group IV) vectors. The spatial distribution of cardiac gene transfer following injection via the transcutaneously inserted catheter (see Materials and Methods) has been previously reported [24], [34]. Intramyocardial delivery of sh-1554 reduced (at day 2 post-transfection) the expression of endogenous *myocd*-*A* and *myocd*-*B* mRNAs by 75% and 60%, respectively, which, in turn, decreased the level of the *myh11* mRNA (40%) (Fig. 4A). In addition, this silencing of *myocd* mRNA expression resulted in a marked decrease of protein levels of MYOCD itself, as well as, of MYH11, ACTA2, and PLN in sh-1554-transfected compared to sh-scb1-transfected myocardium (Fig. 4B). These inhibitory effects seem to be selective because no changes were detected for TNNI, CASQ2, and GAPDH in sh-1554 *versus* sh-scr1 transfections. The most unexpected (and probably functionally relevant) finding was that LV-ESP (an accepted marker for end-systolic performance) was slightly but significantly increased in sh-1554-transfected piglets as compared to controls (Fig. 4C).

On the basis of results obtained from cell-based and *in vivo* test-systems, the interfering sh-1554 vector was chosen as the most promising shRNA for silencing *myocd* expression in diastolic failing heart *in vivo*.

### Inhibition of upregulated myocardin expression in diastolic failing heart results in partial restoration of cardiac function

To test whether inhibition of MYOCD signaling could influence the evolution of diastolic dysfunction in the piglet model of DHF, the following experimental design was used (see Fig. 1A). First, 8-day-old neonatal piglets were injected with Dox or PBS (Group V, VI, and VII). At day 13 after the injection, Dox-treated animals were separated in two groups designated to receive intramyocardial injections of sh-1554 (i.e., Dox/sh-1554 animals) or sh-scr1 vectors (i.e., Dox/sh-scr1 animals), whereas PBS-treated piglets were intramyocardially injected with sh-scr1 vector (i.e., PBS/sh-scr1 piglets). Molecular and functional consequences of targeted *myocd* inhibition were examined in experimental *versus* control groups two and seven days after transfection.

**Figure 5:**
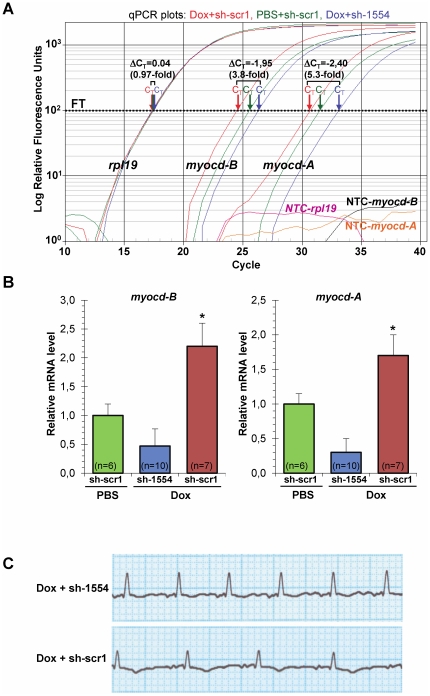
Inhibition of endogenous myocardin expression in failing myocardium of Dox-injected piglets two days after *myocd*-RNAi-vector delivery. (A) Representative qPCR analysis of endogenous *myocd-A* and *myocd-B* expression in failing LV-myocardium transfected with *myocd* silencing (Dox+sh-1554; blue plots) or scrambled (Dox+sh-scr1; red plots) vectors, and in non-failing LV-myocardium transfected with sh-scr1 vector (PBS+sh-scr1; green plots). *Myocd* mRNA levels (normalized to the internal standard *rpl19*) were measured in three LV-samples, each derived from an individual animal. C_T_ - cycle threshold; ΔC_T_ - differences in threshold cycles for target and reference. Calculated fold-change in *myocd* expression is shown. FT - fluorescence threshold. NTC - non-template controls. (B) Average relative decrease in the *myocd-A/B* transcript level in failing myocardium transfected with sh-1554 (blue) vector as compared to that in failing (red) and non-failing (green) myocardium transfected with sh-scr1 vector. *p≤0.05. # Not significant. (C) Representative ECG recordings (AVL lead) from Dox-injected piglets intramyocardially transfected by *myocd* silencing (Dox+sh-1554) or scrambled (Dox+sh-scr1) vectors.

**Figure 6:**
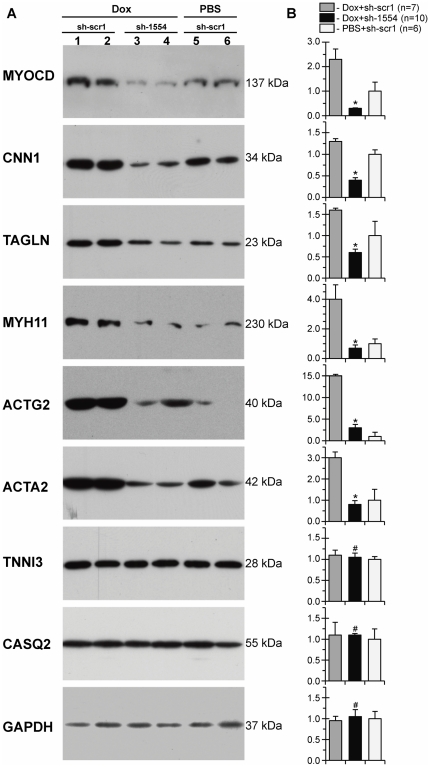
Lowering levels of SM-marker proteins in failing myocardium of Dox-injected piglets two days after *myocd*-RNAi-vector delivery. (A) Representative Western blots of MYOCD, CNN1, TAGLN, MYH11, ACTG2, ACTA2, TNNI3, CASQ2, and GAPDH in failing LV-myocardium transfected with scrambled (Dox+sh-scr1; lane 1, 2) or *myocd* silencing (Dox+sh-1554; lane 3, 4) vectors, and in non-failing LV-myocardium transfected with sh-scr1 vector (PBS+sh-scr1; lane 5, 6). Each LV-sample (1–6) was derived from an individual animal. MW values (kDa) of the bands detected are shown. (B) Overall relative levels of SM-marker proteins in LV-samples as based on average values from each group studied. *p≤0.05. # Not significant.

On day 2 post-delivery, MYOCD knockdown resulted in a significant decrease in the level of both *myocd*-*A* (80%) and *myocd*-*B* (75%) mRNAs in failing LV-myocardium of the Dox/sh-1554 piglets as compared to that in the Dox/sh-scr1 animals (Fig. 5A). In addition, the resulting level of *myocd* transcript expression in failing myocardium of the Dox/sh-1554 group was lower (50% the average decrease) than that in the control PBS/crb1 group (Fig. 5B). Immunoblot analysis confirmed the *myocd*-specific downregulation observed at the mRNA level: the MYOCD protein content was the lowest in the Dox/sh-1554 group as compared to controls (Fig. 6).

Next we measured expression of *myocd*-dependent SM-marker genes in Dox-failing myocardium transfected with *myocd*-silencing vector (see Fig. 6). Compared with Dox-failing myocardium transfected with scrambled vector (Dox/sh-scr1), protein levels were highly decreased for CNN1, TAGLN, MYH11, ACTG2 and ACTA2 in failing myocardium transfected with *myocd*-silencing vector (Dox/sh-1554 animals). Even more importantly, the reduced levels of these SM-marker proteins in Dox/sh-1554 piglets became comparable to those in control animals (PBS/sh-scr1 group). These effects of *myocd* silencing were not the consequence of a general, non-specific impairment of protein expression in transfected myocardium, because the protein content of TNNI, CASQ2 and GAPDH was equal in all groups studied.

To assess the effect of *myocd* silencing on heart function, we used hemodynamic, ECG and intracardiac pressure measurements 2 days after transfection (Table 4). Elevated LV-EDP values, characteristic of impaired diastolic function in the Dox/scr1 group, were significantly decreased in failing hearts transfected with *myocd*-silencing vector (Dox/sh-1554 group) to values similar to those in hearts of PBS-treated piglets (PBS/sh-scr1 group). The re-establishment of impaired diastolic function was associated with lowering the rate of ECG-indicators of myocardial ischemia (ST/T depression, decreased QRS voltage) as well as with the trend toward amelioration of bradycardia in Dox/sh-1554 compared to Dox/sh-scr1 transfected piglets (Fig. 5C). In addition, and in concordance with the above stated evidence (see Fig. 4C), *myocd* silencing resulted in slightly augmented LV-ESP values (i.e., systolic performance) in Dox-failing piglets (see Table 4). Hemodynamic measurements were not significantly different between groups with the exception of slightly higher heart rhythm and lower systolic blood pressure values in Dox/sh-1554 compared to control Dox/sh-scr1 and PBS/sh-scr1 group, respectively.

**Table 4:** Cardiac parameters of piglets injected with Dox or PBS followed by intramyocardial delivery of *myocd*-specific or scramble short-hairpin plasmids.

Parameter	on day 2 post-delivery			on day 7 post-delivery	
	PBS+sh-scr1	Dox+sh-scr1	Dox+sh-1554	Dox+sh-scr1	Dox+sh-1554
Transfected animals, n	6	8	10	8	8
Survived animals, n	6	7	10	4	8
HR, beats/min	124±5	106±4*	136±5	123±10	130±6
Systolic BP, mm Hg	69.0±1.4	61.5±2.0	55.8±3.5	71.5±5.6	68.0±1.6
Diastolic BP, mm Hg	47.0±3.0	44.0±1.5	40.8±3.2	57.0±4.6†	46.0±2.1
H/B ratio ×1000	6.7±0.1	7.3±0.3	7.7±0.3	6.9±0.6	6.8±0.3
LVES pressure, mm Hg	71±1.0	61±4.5*	74±2.6	57.5±3.5†	66.8±2.4
LVED pressure, mm Hg	8.5±1.3	13.0±0.2*	9.5±1.2	12.5±1.2	12.3±0.9
ST/T depression, %	0	100	50	100	80
Low-voltage QRS, %	0	100	20	100	40
LV BNP mRNA, relative value	1.0±0.3	6.2±0.8*	3.7±0.6	7.7±1.2	6.7±1.4

PBS - phosphate-buffered saline; Dox - doxorubicin; sh-scr1 - scramble shRNA expression plasmid 1; sh-1554 - myocardin-specific shRNA expression plasmid; HR - heart rhythm; BP - blood pressure; H/B - heart/body ratio; LVES and LVED - left ventricle end systolic and diastolic pressure values, respectively; ST/T and ORS - ECG values (leads I, II, and AVL). p≤0.05: Dox+sh-scr1 *versus* Dox+sh-1554, 2 days (*) and 7 days (†) post-delivery.

Taken together, the data demonstrated that at an early time point (on day 2 post-delivery), *myocd* inhibition improves functioning of failing hearts. At this time point, we also observed a decline in BNP mRNA expression in failing LV-myocardium transfected with *myocd*-silencing vector compared to controls (see Table 4).

**Figure 7:**
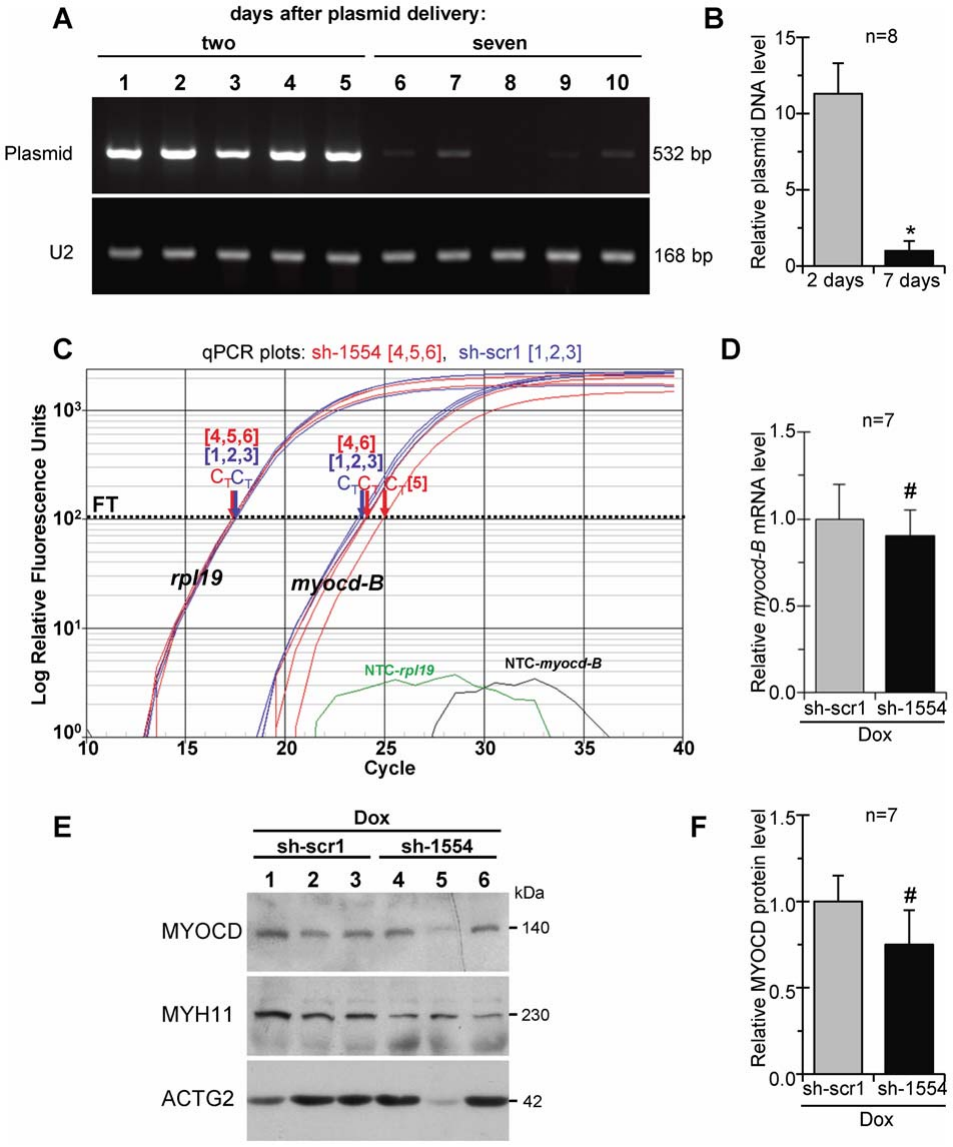
Recovery of endogenous myocardin expression in failing myocardium of Dox-injected piglets 7 days after *myocd*-RNAi-vector delivery. (A) Representative ethidium bromide-stained gel showing detection of plasmid DNA in LV-samples harvested from Dox-failing piglets two (lane 1–5) and six (lane 6–10) days after intramyocardial delivery of *myocd*-silencing (sh-1554) plasmid vector. U2 gene expression was used as an internal standard. (B) Relative levels of plasmid DNA in LV-samples as based on average values from each group studied. *p≤0.05. (C) Representative qPCR analysis of endogenous *myocd-B* mRNA levels in failing LV-myocardium transfected with *myocd* silencing (sh-1554; red plots) or scrambled (sh-scr1; blue plots) vectors. Other details as in the caption to Fig. 5A. (D) Overall *myocd-B* mRNA levels in sh-1554-transfected as compared with scr1-transfected failing piglets. # Not significant. (E) Representative Western blots of MYOCD, MYH11, and ACTG2 in failing LV-myocardium transfected with scrambled (sh-scr1; lane 1–3) or *myocd* silencing (sh-1554; lane 4–6) vectors. Each LV-sample (1–6) was derived from an individual animal. MW values (kDa) of the bands detected are shown. (F) MYOCD protein levels in LV-samples as based on average values from each group studied. # Not significant.

The efficacy of plasmid-based (non-viral) vectors can be compromised by a rapid degradation of delivered plasmid constructions *in vivo* [38]. In this work, at day 7 post-delivery, the level of plasmid DNA in transfected LV-myocardium was an average 10-fold lower as compared to 2 days after administration, and it was detected at near quantification limit in 3 out of 8 examined piglets (Fig. 7A, B). These results suggested that the degree of plasmid-based silencing of *myocd* expression also drops, leading to the restoration of upregulated levels of *myocd* in failing myocardium at distant time points after transfection.

At day 7 post-delivery, as revealed by qPCR analysis, the endogenous *myocd* mRNA expression in the Dox/sh-1554 group was re-activated (with the exception of one animal; see Fig. 7C, plot 5) to a level comparable to that in the failing control animals that received the scrambled vector (Dox/sh-scr1 group) (Fig. 7D). At the protein level, *myocd* expression in experimental animals was also similar to that of Dox-treated controls showing a reduced MYOCD content in only one of eight Dox/sh-1554 transfected piglets (Fig. 6E, lane 5 and Fig. 6F). Such re-activation of *myocd* expression was associated with an almost full return of ACTG2 and the partial restoration of MYH11 protein levels in failing myocardium of Dox/sh-1554 piglets as compared with those in failing control animals that received the scrambled vector (i.e., Dox/sh-scr1 group; see Fig. 6E).

Subsequent functional analysis of Dox/sh-1554 transfected piglets at day 7 post-delivery demonstrated that a return to elevated *myocd* expression levels is associated with the re-deterioration of diastolic function (elevated LV-EDP values) as compared with the same experimental run at day 2 post-delivery (see Table 4). These results suggested that a reactivation of MYOCD signaling cascade after *myocd* inhibition burst is maladaptive particularly in terms of LV diastolic function. Nevertheless, in spite of a return to elevated *myocd* expression and impaired diastolic function at day 7 after transfection, failing piglets receiving the *myocd*-silencing vector were characterized by higher LV-ESP, less frequency of ischemic ECG-manifestations, and higher survival rate as compared with failing piglets transfected with scrambled vector. Of note, higher LV-ESP values in Dox+sh-1554 *versus* Dox+sh-scr1 groups, both after two and seven days, suggested a beneficial effect of *myocd*-targeting in preserving LV function (see Table 4).

Collectively, the results strongly suggest that a mild inhibition of *myocd*, at advanced stages of experimental DHF, downregulates the activated expression of SM-marker genes in failing LV-myocardium associated with partial restoration of diastolic function and lower premature mortality of DHF-piglets compared to controls.

## Discussion

The association between activated MYOCD signaling and HF has been established in studies of ventricular myocardium in animal models and in human myocardium from patients with end-stage HF undergoing heart transplantation [19], [21]. Pathological forms of ventricular hypertrophy were also found to be associated with an aberrantly elevated expression of *myocd* and with severe downregulation of competitive inhibitors of MYOCD in ventricular myocardium [25]. Moreover, *myocd* is upregulated in blood mononuclear cells of patients with hypertrophic cardiomyopathy [39].

The present work was undertaken in an attempt to integrate upregulated MYOCD signaling into the pathogenesis of HF, using targeted RNAi-mediated *myocd* gene inhibition in the porcine model of DHF. Our model of Dox-induced DHF in neonatal piglets is justifiably suitable for a given context-of-use and task, since in this model the development of diastolic dysfunction is associated with the exaggerated activation of *myocd* and downstream SM-targets in failing hearts (see Fig. 2). Of note, the expression of some SRF-dependent sarcomeric genes (such as ACTC1, MYH7, and TNNI) was not affected in Dox-injected piglets.

To the best of our knowledge, this is the first indication of activation of a SM-gene program in DHF, the pathological condition resulting from increased stiffness and impaired relaxation of ventricular myocardium that is associated with alterations in myofilament and calcium handling proteins (reviewed in [40], [41]).

In this sense, we found that levels of PLN (a key regulator of sarco-endoplasmic reticulum Ca^2+^-ATPase) are elevated in DHF piglets (see Fig. 2). In large animal models, overexpression of PLN in the heart resulted in altered myocardial structure [42] whereas PLN inhibition reversed HF progression [43]. In this work, a mild silencing of *myocd* in healthy neonatal piglets resulted in a concomitant decrease in the expression of MYH11, ACTA2, and PLN that, in turn, was associated with an increase, although not highly significant, of end-systolic performance (see Fig. 4C). Formally viewed, these data are well in line with our previous results that *in vivo* forced expression of *myocd* in LV- myocardium impaired systolic performance in neonatal pig heart [24]. Only further studies will refine these findings and reveal to what extent modulations of *myocd* expression in the fast-growing neonatal myocardium could contribute to cardiac performance.

The moderate reduction (60–70%) of ventricular *myocd* activity observed in these experiments did not lead to any deterioration of cardiac function in neonatal piglets, as one could have expected from previous studies, where cardio-restricted *myocd* knockdown (85%) was found to induce dilated cardiomyopathy and fatal HF in postnatal mice [17]. These significant differences in the cardiac phenotypes of sh-*myocd*-transfected and genetically *myocd*-depleted postnatal hearts can be explained by many factors on multiple levels. As compared to *myocd* gene ablation, a moderate sh-mediated inhibition of *myocd* expression: (1) did not lead to the almost complete loss of the targeted mRNA (i.e., residual expression of *myocd*) in the LV-myocardium and (2) did not affect *myocd* activity in other cardiac chambers (data not shown). In addition, species-specific differences in MYOCD requirements for postnatal LV-remodeling, in pigs as compared to mice, could not be excluded, although the underlying mechanisms remain elusive at present.

Distinct splice variants of *myocd* were reported to be expressed in mammalian heart [13], [19], [44], [45], [46], [47] with the *myocd B* transcript being more abundant in pig ventricular myocardium [24]. In this work, a pool of potential sh-RNAs complementary to different regions of the porcine *myocd-B* mRNA was generated, and after validation in three distinct experimental setups, the sh-interfering RNA1554 was chosen as the most promising sequence for silencing *myocd* in DHF, because it effectively targets the endogenous pool of *myocd-A/B* mRNAs in both pig aortic SM-cells *in vitro* (see Fig. 3B) and normal piglet LV-myocardium *in vivo* (see Fig. 4A).

We explored the potential role of *myocd* inhibition in the piglet heart during HF progression by direct delivery of the *myocd*-specific silencing plasmid (i.e., sh-1554 plasmid, see Fig. 1C) into failing LV-myocardium. Comparative analysis of gene expression and functional consequences of *myocd* silencing at different time intervals (i.e., on day 2 and 7 after delivery; see Table 4) revealed the following noteworthy observations: (1) the early moderate inhibition of endogenous *myocd* expression attenuated expression of SM-marker genes in failing LV-myocardium, (2) this decrease of MYOCD signaling activity in failing myocardium to the level comparable with that in non-failing animals resulted in improvement of impaired diastolic function and amelioration of myocardial ischemic conditions, (3) the posterior restoration of elevated *myocd* expression led to activation of *myocd*-dependent SM-marker genes in failing LV-myocardium associated with a return to altered diastolic function, and (4) the transient inhibition of MYOCD signaling at advanced stages of DHF delayed the progression of diastolic dysfunction and extended the survival of failing piglets. In rats, myocardial ischemia and bradycardia are two ECG-manifestations of the acute Dox-induced cardiotoxicity, embodied with decreased heart rhythm [48]. In our model of Dox-induced DHF, *myocd* targeting was associated with amelioration of such ECG-manifestations suggesting an involvement of MYOCD signaling in the regulation of cardiac function.

It is not unreasonable to suggest that impaired diastolic function in Dox-treated piglets is conditioned, at least in part, by overexpression of SM-marker genes (CNN1, TAGLN, MYH11, ACTG2, and ACTA2) in failing myocardium. The induction of these SM-marker genes seems not to be associated with activation of *srf* expression because *srf* transcript levels were not changed in failing as compared to non-failing piglet myocardium. Thus, it is tempting to speculate that the activation of SM-marker genes in DHF myocardium can be attributed, at least in part, to selective upregulation of MYOCD. In cardiomyocytes, the level and functional activity of MYOCD is regulated by a balance of positive [21], [22], [49] and negative [25], [50], [51], [52] factors. In this sense, our present results can be interpreted as suggesting that failing ventricular myocardium is characterized by impaired counterbalance of MYOCD factors resulting in the overexpression of SM-marker genes, a functionally maladaptive response [53].

*Myocd* overexpression correlates with a wide array of pathological conditions. Upregulation of *myocd* enforces cardiac hypertrophic response [21], [22], [25], antagonizes cell proliferation in growth- and tumor-related processes [54], [55], [56], and in some situations promotes hypercontractile vascular phenotype that may contribute to neurovascular dysfunction [57], [58]. Given that elevated expression of *myocd* is a frequent event in HF conditions [19], [21], [25], our results highlight the benefits of inhibiting the upregulated MYOCD signaling pathway in failing ventricular myocardium: a moderate *myocd* suppression was sufficient to downregulate the elevated expression of MYOCD-dependent SM-marker genes, ameliorate diastolic chamber dysfunction, and prevent pre-mature mortality of DHF animals.

More broadly, the results of the present work give a clear indication that adjusting altered *myocd* expression to the range of physiological variation could be essential in order to reduce and normalize the expression of SRF/MYOCD-dependent SM-marker genes, that are upregulated in advanced HF of diverse etiologies ([27], [28], [29]; this work), without compromising the physiological functions of MYOCD signaling [17] as a part of the adaptive response of the heart to stress.
